# Stage‐dependent management paradigms for MEN1‐related Zollinger–Ellison syndrome: A vignette‐based study by the ENETS MEN1 Task Force across 57 expert centers

**DOI:** 10.1111/jne.70304

**Published:** 2026-11-09

**Authors:** Kosmas Daskalakis, Carolina R. C. Pieterman, Detlef K. Bartsch, Jerena Manoharan, Simona Grozinsky‐Glasberg, James R. Howe, Mikkel Andreassen, Nehara Begum, Dermot O'Toole, Timm Denecke, Antongiulio Faggiano, Massimo Falconi, Stefano Partelli, Andreas Pascher, Staffan Welin, Els Nieveen van Dijkum, Alia Munir, Aurel Perren, Sara Massironi, Ana P. Santos, Amit Tirosh, Anna Caterina Milanetto, Roberta Modica, Agnieszka Stefańska, Guenter Kloeppel, Piero Ferolla, Gerlof D. Valk, Gregory Kaltsas

**Affiliations:** ^1^ Second Department of Surgery Korgialenio‐Benakio Red Cross General Hospital Athens Greece; ^2^ Department of Endocrine Oncology University Medical Center Utrecht Utrecht the Netherlands; ^3^ Department of Visceral, Thoracic and Vascular Surgery Philipps‐University Marburg Marburg Germany; ^4^ Neuroendocrine Unit, ENETS Center of Excellence, Division of Medicine Hadassah Medical Center and Faculty of Medicine, The Hebrew University Jerusalem Israel; ^5^ University of Iowa Carver College of Medicine Iowa City Iowa USA; ^6^ Department of Nephrology and Endocrinology Copenhagen University Hospital Rigshospitalet Copenhagen Denmark; ^7^ Department of General, Visceral, Thoracic and Endocrine Surgery Johannes Wesling Klinikum Minden, University Hospital of the Ruhr‐University Bochum (RUB) Minden Germany; ^8^ Department of Clinical Medicine St James Hospital, Trinity College Dublin & National Centre for Neuroendocrine Tumours, St Vincent's University Hospital Dublin Ireland; ^9^ Klinik für Diagnostische und Interventionelle Radiologie Universitätsklinikum Leipzig Leipzig Germany; ^10^ Endocrinology Unit, Department of Clinical and Molecular Medicine Sant'Andrea Hospital, ENETS Center of Excellence, Sapienza University of Rome Rome Italy; ^11^ Pancreatic and Transplantation Surgical Unit IRCCS San Raffaele Hospital, Università Vita‐Salute Milan Italy; ^12^ Klinik für Allgemein, Viszeral und Transplantationschirurgie Universitätsklinikum Münster Münster Germany; ^13^ Endocrine Oncology, Department of Medical Sciences Uppsala University Uppsala Sweden; ^14^ Department of Surgery, Cancer Center Amsterdam University of Amsterdam Amsterdam the Netherlands; ^15^ Sheffield Teaching Hospitals NHS Foundation Trust Sheffield UK; ^16^ Institute of Tissue Medicine and Pathology University of Bern Bern Switzerland; ^17^ Vita‐Salute San Raffaele University Milan Italy; ^18^ Istituti Ospedalieri Bergamaschi Bergamo Italy; ^19^ Endocrinology Department Portuguese Oncology Institute of Porto (IPO Porto) Porto Portugal; ^20^ Porto Comprehensive Cancer Center Raquel Seruca (P.CCC) Porto Portugal; ^21^ Precancerous Lesions and Early Cancer Management (PRECAM) Group|IPO Porto Research Center (CI‐IPOP) Porto Portugal; ^22^ Division of Endocrinology, Metabolism, and Diabetes, Endocrine Neoplasia Translational Research Center Sheba Medical Center Ramat Gan Israel; ^23^ UniCamillus International Medical University Rome Italy; ^24^ Department of Surgery Policlinico Abano Abano Terme Italy; ^25^ Endocrinology, Diabetology and Andrology Unit, Department of Clinical Medicine and Surgery Federico II University of Naples Naples Italy; ^26^ Department of Endocrinology, Endocrine Oncology, Nuclear Medicine and Internal Diseases University Hospital in Krakow, ENETS Center of Excellence Krakow Poland; ^27^ Institute of Pathology Technical University of Munich Munich Germany; ^28^ NET Multidisciplinary Group Umbria Regional Cancer Network Perugia Italy; ^29^ First Propaedeutic Department of Internal Medicine National and Kapodistrian University of Athens Athens Greece

**Keywords:** gastrinoma, multiple endocrine neoplasia type 1, neuroendocrine tumor, Zollinger–Ellison syndrome

## Abstract

Management of Zollinger–Ellison syndrome (ZES) in patients with multiple endocrine neoplasia type 1 (MEN1) remains controversial, particularly in non‐metastatic disease where the role of surgery is debated. In contrast, treatment of metastatic disease is guided by established systemic approaches. We aimed to evaluate stage‐dependent clinical decision‐making in MEN1‐related ZES using standardized clinical vignettes across ENETS Centers of Excellence (CoEs). A questionnaire‐based study using two clinical vignettes was conducted by the ENETS MEN1 Task Force across all 68 ENETS CoEs, with a predefined participation target of 70%. Additional non‐ENETS expert centers were also invited. Dedicated multidisciplinary teams provided institutional consensus responses. Scenarios included non‐metastatic disease, locally metastatic disease, and distant metastatic disease. Centers were stratified by volume using a cutoff of 30 MEN1 patients in active care. Data were analyzed descriptively, comparing management strategies between low‐ and high‐volume centers. Fifty‐seven centers participated (52 of 68 ENETS CoEs [76.5%] and 5 additional non‐ENETS expert centers). All 57 centers completed the questionnaire in full. All cases received symptomatic therapy with proton pump inhibitors. In non‐metastatic disease, management was heterogeneous, with 56.1% recommending surgery. Centre volume strongly influenced decisions (73.1% vs. 41.9% surgery in low‐ vs. high‐volume centers, *p* = .018). Preferred surgical approaches included duodenotomy with regional lymphadenectomy (43.9%) and the Thompson procedure (left pancreatectomy, duodenal exploration with excision of tumors and node dissection, enucleation of pancreatic head lesions; 26.3%). In locally metastatic disease, surgical management increased to 78.9%, with a shift toward more extensive procedures including pancreaticoduodenectomy (35.1%). In distant metastatic disease, somatostatin analogues were the systemic therapy of choice (50.9%), followed by curative‐intent surgery with pancreaticoduodenectomy or duodenotomy with regional lymphadenectomy combined with liver resection or thermal ablation of liver lesions (35.1%), while no other systemic treatments were selected by any center. A liver biopsy was recommended by 43.9% of centers. Management of MEN1‐related ZES demonstrates stage‐dependent convergence. In early disease, decision‐making—particularly regarding surgical versus medical management—is highly variable and influenced by center experience, whereas advanced disease shows more consistent, guideline‐aligned treatment patterns. The high participation rate across ENETS CoEs suggests that these findings are likely to be relevant to expert neuroendocrine tumor practice.

## 1 INTRODUCTION

Zollinger–Ellison syndrome (ZES) caused by duodenal‐pancreatic (dp) NETs represents one of the most clinically challenging manifestations of multiple endocrine neoplasia type 1 (MEN1).^1^, ^2^ MEN1 is caused by inactivating mutations of the MEN1 gene. This is associated with a lifetime risk of developing dpNETs exceeding 70%.^1^, ^3^, ^4^ Advanced dpNETs remain a leading cause of premature mortality in MEN1, accounting for 40–45% of disease‐related deaths.^1^, ^5^, ^6^, ^7^ While the biochemical diagnosis of ZES is well established, its management in the context of MEN1 remains complex and is not yet standardized.^2^, ^4^ A key challenge in MEN1‐related ZES is the unique biological behavior of gastrinomas that are typically small, multifocal tumors almost ever located in the duodenum rather than the pancreas.^1^, ^8^ Lymph node metastases are frequently observed in MEN1‐associated duodenopancreatic NETs, including gastrinomas, although their reported incidence varies widely across surgical series and does not consistently appear to adversely affect survival. In contrast, distant metastases of dpNETs, occurring in approximately one quarter of patients in larger cohorts, are a major determinant of MEN1‐related mortality.^9^, ^10^ MEN1‐related gastrinomas are often submucosal and difficult to detect with conventional imaging, endoscopic ultrasonography, and/or functional imaging.^11^, ^12^ Furthermore, the presence of multiple synchronous lesions complicates surgical planning and limits the likelihood of complete tumor eradication in case of local excision.

The role of surgery in non‐metastatic MEN1‐related ZES remains particularly controversial with some advocating early surgical intervention aiming to prevent lymph node and liver metastases, whereas others favor a more conservative management.^3^, ^13^ Surgical approaches range from limited duodenotomy with excision of gastrinomas and lymphadenectomy to more extensive procedures, including pancreas‐preserving total duodenectomy with peripancreatic lymph node dissection for duodenal lesions, pancreaticoduodenectomy or combined resections.^14^, ^15^ However, these interventions carry significant morbidity but provide long‐term cure in about 80% of patients in newer studies.^1^, ^7^, ^14^, ^16^ Conversely, a more conservative strategy involving medical management with proton pump inhibitors (PPIs) with or without somatostatin analogues (SSAs), combined with active surveillance, is increasingly adopted.^2^ However, the long‐term impact of delaying surgical intervention remains uncertain. Management of metastatic MEN1‐related dpNETs is largely guided by trials supporting SSAs, everolimus, sunitinib, PRRT, and cabozantinib in broader NET populations.^17^, ^18^, ^19^, ^20^, ^21^, ^22^ However, these studies did not establish efficacy specifically in MEN1‐associated gastrinomas, and treatment decisions therefore rely on extrapolated evidence. Surgical intervention in this setting is typically reserved for highly selected patients with limited metastatic burden or for symptom control.^23^

Despite the availability of international guidelines from European Neuroendocrine Tumor Society (ENETS), National Comprehensive Cancer Network (NCCN), and other expert groups, significant practice variation persists even among centers of excellence.^1^, ^3^, ^16^, ^24^ The 2025 MEN1 guidelines explicitly acknowledge that no definitive recommendation can be made regarding the timing and extent of surgery for gastrinomas, and that decisions should be made by multidisciplinary teams through shared decision‐making.^1^ The 2023 ENETS guideline stated that the timing and type of surgery for MEN1‐related ZES should be individualized according to the patient's characteristics (e.g., age, comorbidities, pre‐existing insulin‐dependent diabetes) and preferences.^16^ This variability likely reflects differences in institutional experience, interpretation of limited evidence, and access to specialized care. To address these uncertainties, and specifically to elucidate how clinical decisions are made in areas lacking consensus, the European Neuroendocrine Tumor Society MEN1 Task Force designed a vignette‐based study across ENETS Centers of Excellence (CoE). By presenting standardized clinical scenarios reflecting increasing disease burden—ranging from non‐metastatic to locally advanced and distant metastatic MEN1‐related ZES—we aimed to systematically evaluate real‐world clinical decision‐making, examine how management strategies evolve across disease stages, and identify key drivers of variation in practice.

## 2 METHODS

### 2.1 Study design and center recruitment

This study was an initiative of the ENETS MEN1 Task Force and conducted as a vignette‐based questionnaire distributed to all 68 ENETS CoEs. A predefined participation target of 70% was established to ensure adequate representation of expert neuroendocrine tumor (NET) practice. In addition to the 52 participating ENETS CoEs, five non‐ENETS expert centers were identified and invited through ENETS CoEs' directors and members of the ENETS MEN1 Task Force, based on their clinical expertise in MEN1 management. All five invited centers participated (5/5, 100%). The median number of MEN1 patients in active care at these centers was 16 (range 6–30). A sensitivity analysis excluding the five non‐ENETS centers was performed to assess their potential influence on the study findings.

The questionnaire was developed by the ENETS MEN1 Task Force on previously published ENETS survey methodologies and adapted to focus on clinical dilemmas in MEN1‐related ZES.^25^ Before distribution, the questionnaire was reviewed internally by members of the task force to ensure clarity and clinical relevance. The final questionnaire was distributed electronically via REDCap between May 2025 and March 2026. Reminder invitations were sent to non‐responding centers at predefined intervals. Participating centers were invited to complete the questionnaire on behalf of their multidisciplinary team (MDT). Centers were instructed to discuss each case collectively and submit a single response reflecting institutional consensus. The date of MDT discussion was recorded for each center.

### 2.2 Clinical vignettes

The questionnaire consisted of two clinical vignettes designed to represent different stages of MEN1‐related ZES. Representative imaging from both vignettes is provided in Data S1, Supporting Information along with the complete questionnaire.

Vignette 1 described a 35‐year‐old normocalcemic ZES patient with genetically confirmed MEN1. Work‐up with gastroduodenoscopy, endoscopic ultrasound (EUS), Gallium‐68 DOTA–conjugated Positron Emission Tomography/Computed Tomography (^68^Ga‐DOTA‐PET/CT), and abdominal Magnetic Resonance Imaging (MRI) revealed three pancreatic NETs in the body and tail (5–15 mm in size), two 1‐cm submucosal duodenal lesions, and no evidence of lymph node or distant metastases; biopsy of one duodenal lesion revealed a grade 1 NET positive for gastrin. A subsequent modification of this vignette introduced two ^68^Ga‐DOTA‐PET/CT‐positive lymph nodes near the pancreatic head, representing local lymph node metastatic disease.

Vignette 2 described a 39‐year‐old patient with genetically proven MEN1 under regular surveillance with multiple but stable in size pancreatic NETs (the largest 17 mm deep in the pancreatic head and two 5 mm in the body/tail) along with a small duodenal submucosal lesion. On repeated surveillance imaging, two liver lesions (7 and 12 mm in segment 7) were detected. A ^68^Ga‐DOTA‐PET/CT showed uptake confluent to the liver lesions, the known duodenopancreatic lesions, and two PET‐positive peripancreatic lymph nodes.

### 2.3 Questionnaire structure

The questionnaire followed a structured clinical pathway. For each vignette, participants were asked to select initial management strategies from predefined options, followed by questions addressing different surgical approaches, the impact of lymph node involvement, treatment of metastatic disease, liver biopsy decisions, follow‐up strategies, imaging modalities, determination of primary tumor origin, and histopathological evaluation. Multiple‐choice questions allowed for selection of predefined options, and free‐text fields were included for additional comments.

### 2.4 Data collection and analysis

Data were collected using REDCap electronic data capture tools. Responses were anonymized, and each submission represented one center. Data were exported for analysis using IBM SPSS Statistics. Descriptive statistics were used to summarize responses. Categorical variables were reported as frequencies and percentages. To explore the influence of institutional experience, centers were stratified according to the number of MEN1 patients in active care using a cutoff of 30 patients, corresponding to the median of the cohort, thereby allowing balanced comparison between lower‐ and higher‐volume institutions. Centers with fewer than 30 patients were classified as low‐volume, and those with 30 or more as high‐volume. Chi‐square tests (or Fisher's exact test when expected cell counts were <5) were used to compare management strategies between low‐ and high‐volume centers. A two‐sided *p*‐value of .05 was considered statistically significant. Given the exploratory nature of the study, no correction for multiple comparisons was applied, and results should be interpreted accordingly.

## 3 RESULTS

### 3.1 Centre participation and characteristics

Of the 68 ENETS CoE invited, 52 participated in the study (76.5%), exceeding the predefined participation target of 70%. An additional 5 non‐ENETS expert centers with a median of 16 MEN1 patients in active care (range: 6–30) also participated, yielding a total of 57 participating centers. All 57 centers completed the questionnaire in full (100% completion rate). The number of MEN1 patients in active care varied substantially across centers, with a median of 30 patients (interquartile range 14–53.5; range 2–185; mean 40.2 ± 37.6; Figure S1). A total of 26 low‐volume and 31 high‐volume centers were included following dichotomization using a cut‐off of 30 patients, corresponding to the median number of patients across all participating centers. The median number of patients in active care per center was 12 (range: 2–25) for low‐volume centers and 52 (range: 30–185) for high‐volume centers. Only a small subset of centers (*n* = 5) reported more than 100 patients in active follow‐up.

### 3.2 Multidisciplinary team composition

Multidisciplinary team composition was consistent across centers (Figure S2). Surgeons were present in 94.7% of MDTs (54/57), endocrinologists in 89.5% (51/57), pathologists in 86% (49/57), radiologists in 86% (49/57), nuclear medicine physicians in 84.2% (48/57), oncologists in 84.2% (48/57), and gastroenterologists in 70.2% (40/57).

### 3.3 Management of non‐metastatic disease

In the non‐metastatic scenario (Vignette 1, initial presentation), management strategies demonstrated marked heterogeneity. Surgical intervention was recommended by 32 of 57 centers (56.1%), while 10 (17.5%) selected medical treatment with PPI and SSA initiation, 13 (22.8%) favored PPI alone with active surveillance, and 2 centers (3.5%) selected other approaches, including endoscopic resection (Figure 1A). When stratified by center volume, substantial differences emerged. Low‐volume centers recommended surgical management in 73.1% of cases (19/26), compared with 41.9% (13/31) in high‐volume centers (*χ* ^2^, *p* = .018). High‐volume centers more frequently selected non‐operative management (58.1% vs. 26.9%), including PPI along with SSA treatment (25.8% vs. 7.7%) or PPI alone with active surveillance (25.8% vs. 19.2%; Figure 1B).

**FIGURE 1:**
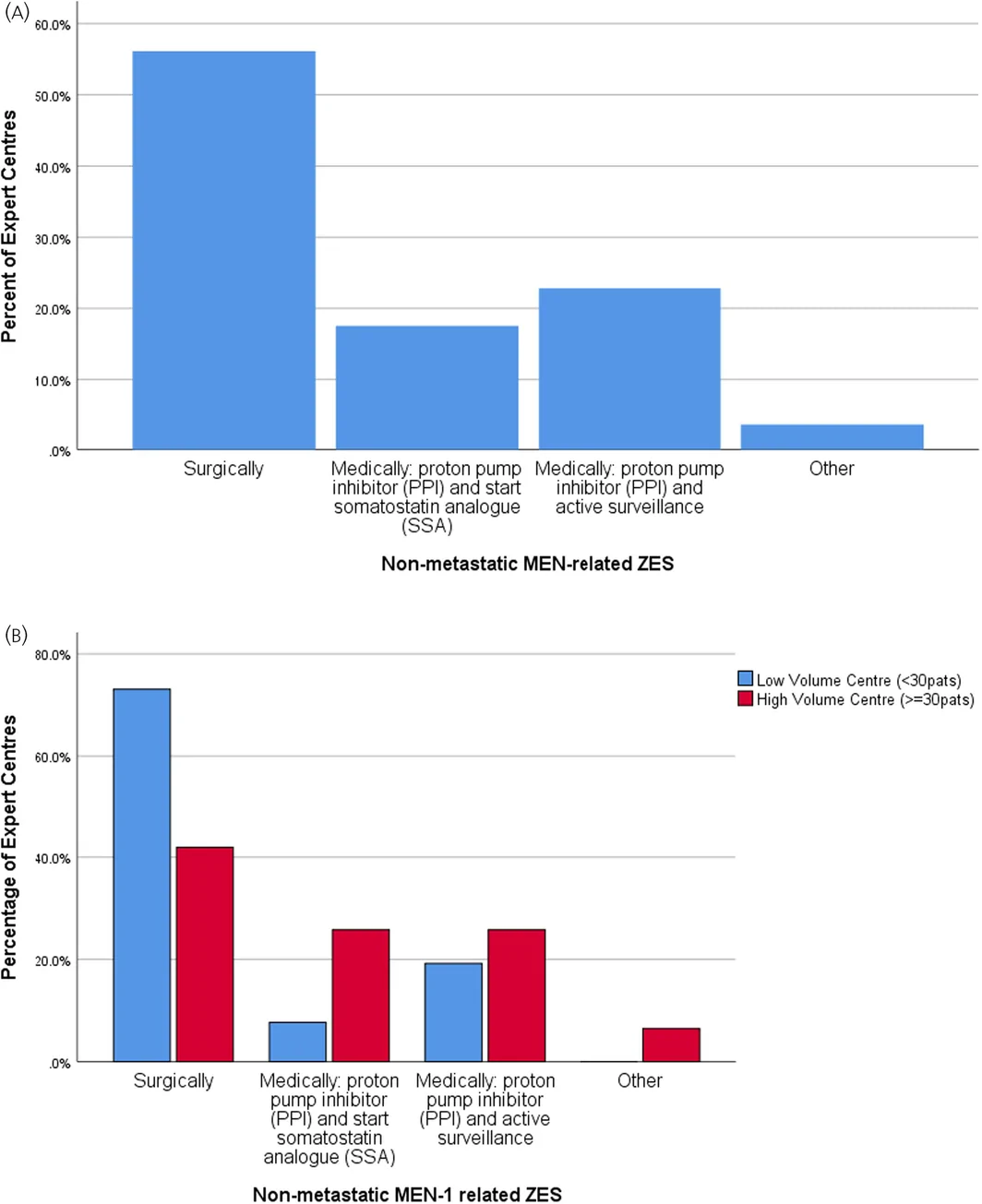
(A) Management of non‐metastatic MEN1‐related ZES (Vignette 1, initial presentation). Bar chart showing the distribution of treatment recommendations across all 57 centers: surgical management (56.1%), proton pump inhibitors (PPI) and somatostatin analogues (SSA: 17.5%), PPI and active surveillance (22.8%), and other (3.5%). (B) Management of non‐metastatic disease stratified by center volume. Grouped bar chart comparing treatment recommendations between low‐volume centers (<30 MEN1 patients, *n* = 26) and high‐volume centers (≥30 patients, *n* = 31). Surgical management was recommended by 73.1% of low‐volume centers versus 41.9% of high‐volume centers (*p* = .018). High‐volume centers more frequently selected non‐operative management (58.1% vs. 26.9%), including PPI along with SSA treatment (25.8% vs. 7.7%) or PPI alone with active surveillance (25.8% vs. 19.2%).

Among all centers, regardless of whether they initially recommended surgery, the preferred surgical approach for non‐metastatic disease was duodenotomy with excision of duodenal NETs/gastrinomas and regional lymphadenectomy, selected by 25 centers (43.9%). The Thompson procedure (left pancreatectomy, duodenal exploration with excision of tumors and node dissection, enucleation of pancreatic head lesions) was selected by 15 centers (26.3%). Pancreaticoduodenectomy (Whipple/Traverso‐Longmire) was selected by 6 centers (10.5%), pancreas‐preserving duodenectomy by 2 (3.5%), and none of the centers selected total duodenopancreatectomy. Nine centers (15.8%) selected other approaches, including 3 endoscopic resections (Figure 2).

**FIGURE 2:**
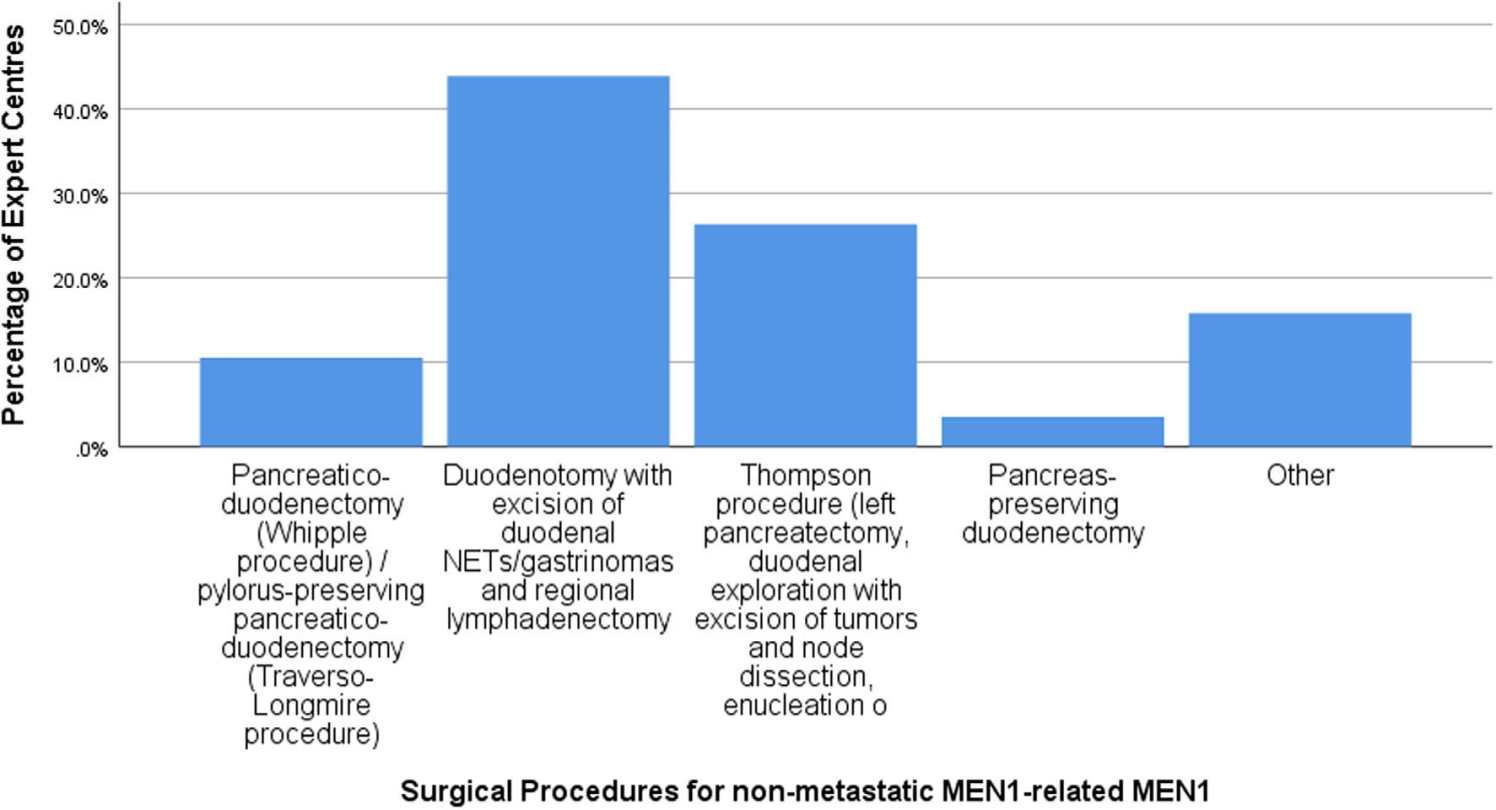
Surgical approach selection in non‐metastatic disease. Bar chart showing the preferred surgical procedure across all centers: duodenotomy with regional lymphadenectomy (43.9%), Thompson procedure (26.3%), pancreaticoduodenectomy (10.5%), and pancreas‐preserving duodenectomy (3.5%). Nine centers (15.8%) selected other approaches, including endoscopic resection.

### 3.4 Management of locally metastatic disease (lymph node metastases)

The introduction of two ^68^Ga‐DOTA‐PET/CT‐positive lymph nodes near the pancreatic head resulted in a clear shift toward surgical intervention. Overall, 45 of 57 centers (78.9%) recommended surgery, while 11 (19.3%) selected PPI with SSA, and one (1.8%) selected PPI with active surveillance (Figure 3). Stratification by center volume revealed that surgical management was recommended by 84.6% (22/26) of low‐volume and 74.2% (23/31) of high‐volume centers (χ^2^, *p* = .336). Although differences between groups persisted, they were less pronounced than in the non‐metastatic scenario.

**FIGURE 3:**
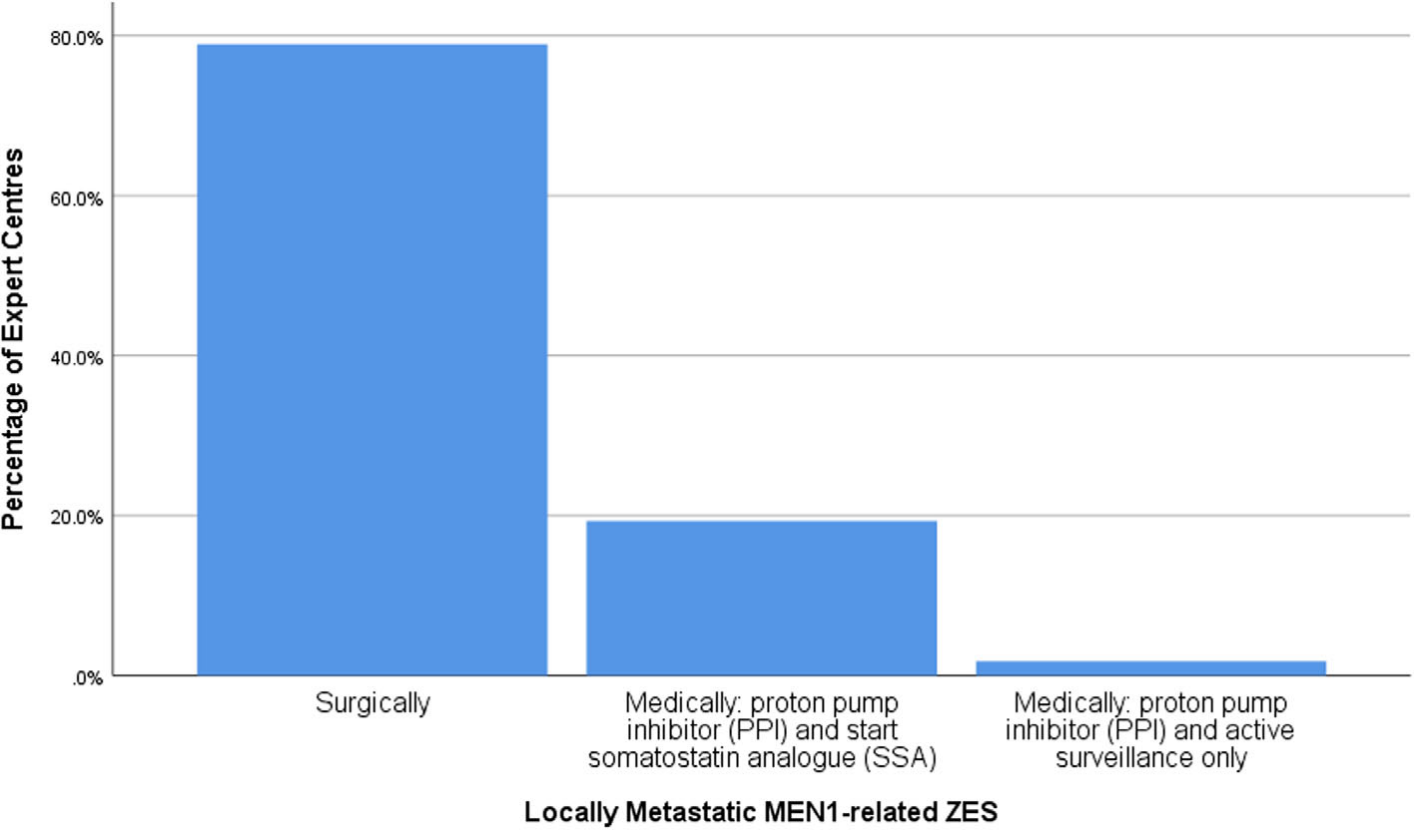
Management of locally metastatic MEN1‐related ZES (lymph node metastases). Bar chart showing the distribution of treatment recommendations when lymph node metastases were introduced: surgical management (78.9%), PPI + SSA (19.3%), and PPI + active surveillance (1.1%).

Pancreaticoduodenectomy (Whipple/Traverso‐Longmire) became the most frequently selected procedure, chosen by 20 centers (35.1%), compared with 10.5% in the non‐metastatic scenario. Duodenotomy with regional lymphadenectomy was selected by 18 centers (31.6%), the Thompson procedure by 8 (14%), total duodenopancreatectomy by 7 (12.3%), and other approaches by 4 centers (7%). None of the centers selected pancreas‐preserving duodenectomy (Figure 4).

**FIGURE 4:**
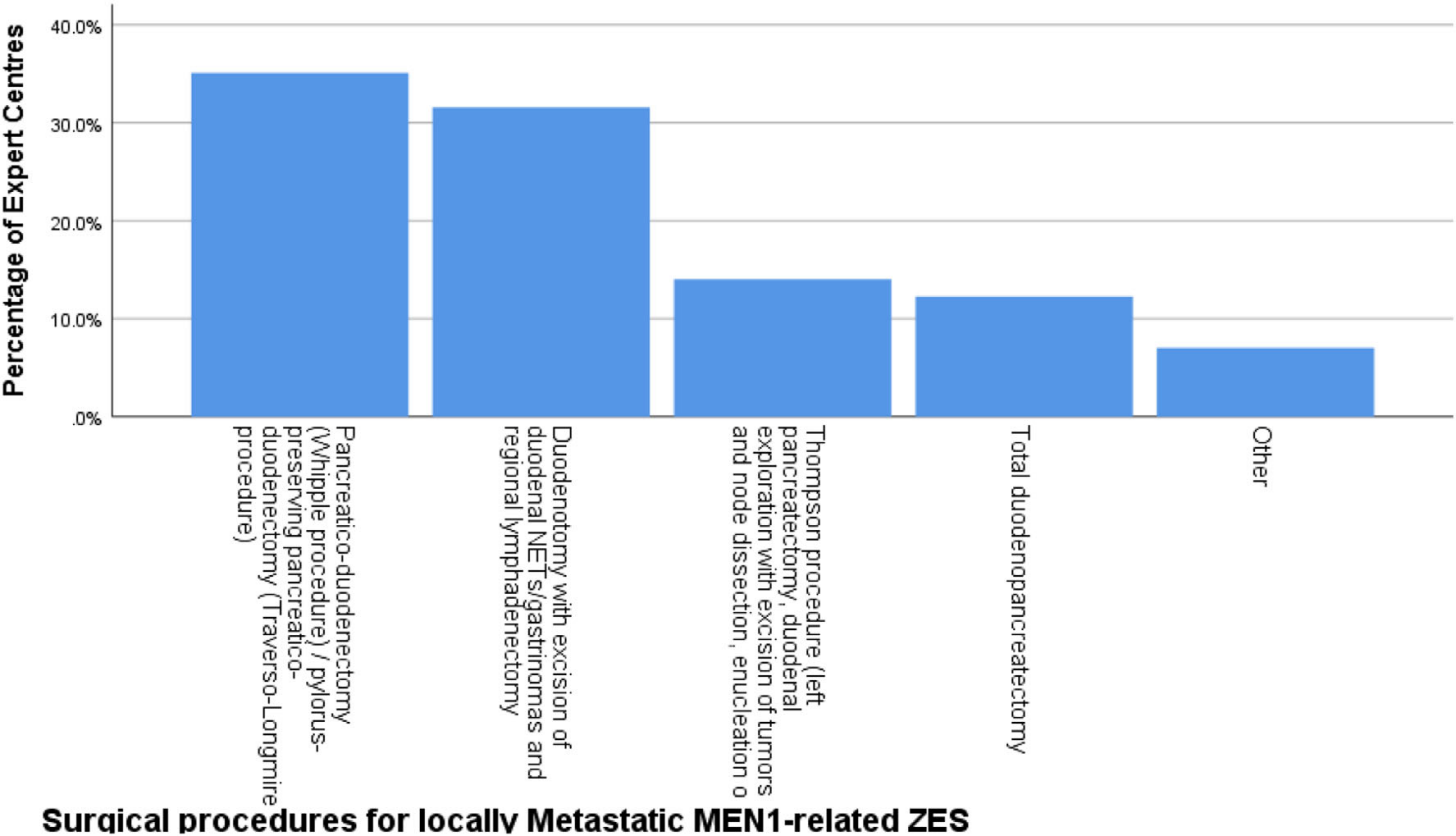
Surgical approach selection in locally metastatic disease. Bar chart showing the preferred surgical procedure when lymph node metastases were present: pancreaticoduodenectomy (35.1%), duodenotomy with regional lymphadenectomy (31.6%), Thompson procedure (14%), total duodenopancreatectomy (12.3%), pancreas‐preserving duodenectomy (0.0%), and other (7%).

### 3.5 Management of distant metastatic disease (liver metastases)

In the presence of distant metastatic disease with liver metastases (Vignette 2), management strategies were more consistent across centers. Somatostatin analogue therapy was the most frequently selected treatment option, chosen by 29 of 57 centers (50.9%). Curative‐intent surgery with pancreaticoduodenectomy and liver resection was selected by 15 (26.3%), and curative‐intent surgery with duodenotomy, gastrinoma excision, lymphadenectomy, and liver resection by 5 centers (8.8%). Active surveillance was selected by 3 (5.3%), and other approaches by 5 centers (8.8%), including thermal ablation in some cases in combination with surgical resection. Notably, neither PRRT nor targeted therapy (everolimus, sunitinib) was selected by any center despite both being offered as response options (Figure 5).

**FIGURE 5:**
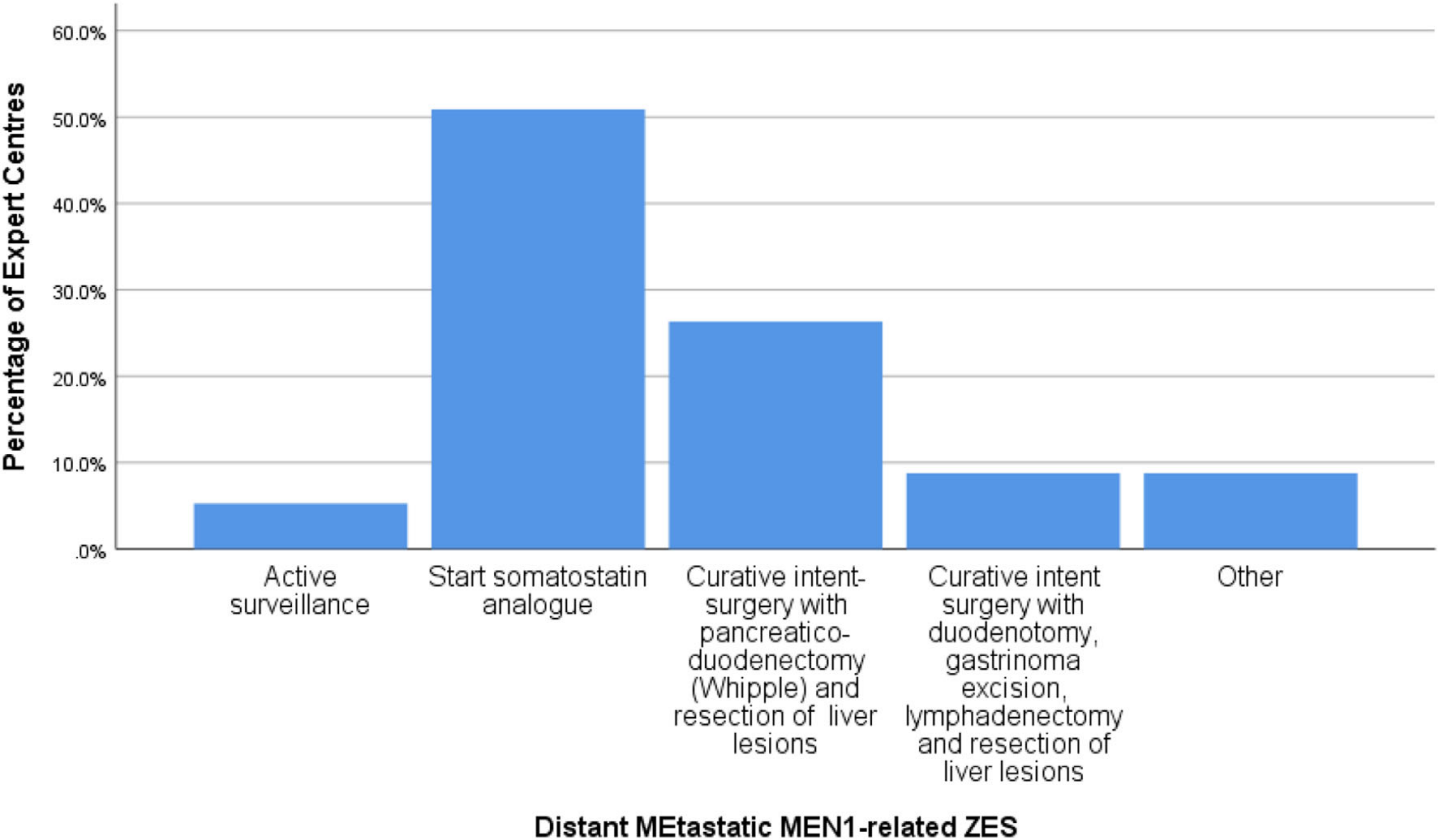
Management of distant metastatic MEN1‐related ZES (Vignette 2, liver metastases). Bar chart showing the distribution of treatment recommendations: somatostatin analogue therapy (50.9%), curative‐intent surgery with pancreaticoduodenectomy and liver resection (26.3%), curative‐intent surgery with duodenotomy‐based approach and liver resection (8.8%), active surveillance (5.3%), PRRT (0.0%), targeted therapy (0.0%), and other (8.8%) including thermal ablation in combination with surgical resection.

When stratified by center volume, similar patterns were observed. Non‐operative management with either SSA or active surveillance alone was recommended by 57.7% (15/26) of low‐volume and 54.8% (17/31) of high‐volume centers (*p* = .830). Curative‐intent surgery with pancreaticoduodenectomy and liver resection was selected by 23.1% (6/26) of low‐volume and 29% (9/31) of high‐volume centers. Curative‐intent surgery with duodenotomy‐based approaches was selected by 15.4% (4/26) of low‐volume and 3.2% (1/31) of high‐volume centers.

### 3.6 Liver biopsy, imaging, and surveillance strategies

When asked whether a liver biopsy would be performed in the metastatic scenario, 25 centers (43.9%) recommended biopsy, while 32 centers (56.1%) did not.

Among centers selecting non‐surgical management, imaging strategies demonstrated a clear preference for cross‐sectional imaging (Figure S5) as MRI of the abdomen was selected by 33 of 50 responding centers (66%), making it the most commonly used modality for follow‐up. Functional imaging with ^68^Ga‐DOTA‐PET/CT was selected by 10 centers (20%), CT by 3 (6%), and other modalities by 4 centers (8%). Neither endoscopic ultrasonography nor FDG‐PET/CT was selected by any center.

Among centers opting for non‐surgical management, surveillance intervals were relatively uniform. Of 49 responding centers, 32 (65.3%) recommended follow‐up every 6 months, 15 (30.6%) selected a 3‐month interval, and two (4.1%) selected another interval. None of the centers selected annual surveillance.

### 3.7 Determination of primary tumor origin

A slight majority of centers (34/57, 59.6%) considered a duodenal gastrinoma the most likely primary source of both lymph node and liver metastases. A further 14 centers (24.6%) considered the duodenal gastrinoma as the most likely primary for lymph node metastases and a non‐functioning pancreatic NET (NF‐PanNET) as the most likely primary for liver metastases. Five centers (8.8%) attributed both lymph node and liver metastases exclusively to a PanNET. No center selected the reverse scenario (non‐functioning PanNET primary for lymph nodes and duodenal gastrinoma for liver metastases). Four centers (7%) selected other possibilities.

### 3.8 Histopathological and immunohistochemical evaluation

#### 3.8.1 Liver biopsy immunohistochemistry

Among centers recommending liver biopsy (*n* = 25), there was a high degree of agreement regarding immunohistochemical evaluation. Gastrin staining was requested by all centers (100%), transcription factor analysis (TTF‐1, Islet‐1, PDX1, ARX, CDX2) by 23 centers (92%), and Somatostatin Receptor 2 (SSTR2) immunohistochemistry by 19 centers (76%). Five centers (20%) requested additional markers, including PITX2, INSM1, and DAXX/ATRX or ALT CISH analysis.

#### 3.8.2 Surgical specimen evaluation

Following surgical resection, standard neuroendocrine markers (synaptophysin/chromogranin A) were assessed universally across specimen types: pancreatic NET (100%), duodenal NET (98.1%), lymph node (92.6%), and liver metastases (92.6%). Ki‐67 proliferation index was similarly assessed across all specimen types (pancreatic NET 100%, duodenal NET 98.2%, lymph node 94.5%, liver metastases 98.2%).

Gastrin immunohistochemistry was widely performed across all specimen types (pancreatic NET 88.2%, duodenal NET 98.0%, lymph node 86.3%, liver metastases 92.2%). DAXX/ATRX assessment was performed by 35 of 57 centers (61.4%), with preferential application to pancreatic NETs (97.1%) compared with duodenal NETs (54.3%), lymph nodes (48.6%), and liver metastases (57.1%). ALT FISH was used by only 15 centers (26.3%), predominantly for pancreatic NETs (100%) with limited application to other specimen types (duodenal NET 33.3%, lymph node 20%, liver metastases 26.7%).

ARX/PDX1 transcription factor analysis was performed by 20 centers (35.1%), again with preferential application to pancreatic NETs (95%) compared with duodenal NETs (45%), lymph nodes (35%), and liver metastases (40%). Insulin and glucagon immunohistochemistry were performed by 26 (47.3%) and 22 (40.0%) centers, respectively, with both markers applied universally to pancreatic NETs (100%) but variably to other specimen types.

### 3.9 Sensitivity analysis

A sensitivity analysis restricted to the 52 ENETS CoEs showed that the principal findings remained largely unchanged after exclusion of the five non‐ENETS centers. Surgery remained the most frequently recommended management strategy for non‐metastatic disease (27/52, 51.9%, versus 32/57, 56.1% in the overall cohort) and locoregional lymph‐node metastatic disease (40/52, 76.9%, versus 45/57, 78.9%). For distant metastatic disease, somatostatin analogues remained the most frequently selected treatment (26/52, 50.0%, versus 29/57, 50.9%). The association between center volume and surgical management in non‐metastatic disease also persisted, with surgery recommended more frequently by low‐volume centers (<30 MEN1 patients) than high‐volume centers (≥30 MEN1 patients; 16/22, 72.7%, versus 11/30, 36.7%; *χ* ^2^ = 6.61, *p* = .010).

## 4 DISCUSSION

This study provides a comprehensive evaluation of clinical decision‐making in MEN1‐related ZES across 57 centers, including 52 of 68 ENETS CoEs (76.5%) and 5 additional non‐ENETS expert centers. The participation rate exceeded the predefined target of 70%, and the 100% completion rate underscores the clinical relevance of the questions addressed and the engagement of the NET community with this initiative. The inclusion of 76.5% of all ENETS CoEs provides a robust and representative sample of expert NET practice. This high participation rate distinguishes the present study from previous surveys in the field, which have typically achieved lower response rates and may therefore be subject to greater selection bias. The consistency of MDT composition across centers confirms that responses reflect genuinely MDT decision‐making rather than single specialty perspectives, as recommended by current guidelines.^1^, ^3^, ^16^, ^24^

The key findings of this study are threefold. First, clinical decision‐making in non‐metastatic disease is highly heterogeneous, particularly regarding the choice between surgical and conservative medical management. Second, center volume significantly influences management strategies, with more high‐volume centers favoring more conservative approaches. Third, increasing tumor burden is associated with progressive convergence toward more standardized, guideline‐aligned treatment strategies, particularly in the diffuse metastatic setting. The marked heterogeneity observed in the non‐metastatic setting is consistent with the complex biological behavior of MEN1‐associated gastrinomas, which are typically small, multifocal, and predominantly located in the duodenum.^1^, ^3^, ^8^ These characteristics limit the likelihood of complete surgical resection and contribute to high recurrence rates, unless more extensive surgical procedures, such as pancreaticoduodenectomy, are performed.^14^, ^26^ At the same time, the variable natural history of MEN1‐related gastrinomas, with many tumors demonstrating relatively indolent behavior, creates a therapeutic dilemma in which clinicians must balance the potential benefits of early intervention against operative morbidity and uncertain long‐term outcomes.^2^, ^4^

The strong influence of center volume on decision‐making aligns with the 2025 expert practice guidelines, which emphasize that no definitive recommendation can be made on decisions regarding the timing and extent of surgery, and therefore these decisions should be made by multidisciplinary teams in centers of expertise and the timing and type of surgery should be individualized according to the patient's characteristics and preferences after counseling.^1^, ^16^ The companion systematic review by English et al. further underscores the low certainty of available evidence, noting that approximately 90% of 3048 publications reviewed were retrospective series with sparse data insufficient to provide high or moderate certainty evidence.^5^ Greater experience with MEN1 may lead to a more conservative strategy, reflecting increased familiarity with the indolent nature of many tumors and the limitations of surgical cure combined with a high complication rate.^27^, ^28^ The absence of prospective randomized trials further contributes to this variability.^4^, ^6^, ^29^

In the recent French AFCE/GTE cohort study, analysis of 233 ZES patients with MEN1 demonstrated that 15‐year survival among non‐metastatic patients was not significantly better in operated patients compared to those managed non‐operatively (82% vs. 70%, *p* = .2), although the high rate of invaded lymph nodes (71%) and absence of operative mortality were cited as indirect arguments for surgery.^30^ This lack of clear survival benefit may explain the conservative approach favored by high‐volume centers in our study. Conversely, others have advocated for surgical intervention over long‐term PPI therapy, arguing that surgery offers the potential for biochemical cure and avoidance of lifelong acid suppression.^13^, ^14^

The stage‐dependent shifts in surgical approach selection are noteworthy. The preference for organ‐preserving approaches in non‐metastatic disease is consistent with contemporary recommendations that parenchymal‐sparing surgery is preferred if feasible.^3^, ^24^ The escalation toward pancreaticoduodenectomy with nodal involvement is supported by data that demonstrated a 82% long‐term cure rate compared with 16–25% for non‐pancreaticoduodenectomy approaches.^14^ Similarly, 92% of patients undergoing pancreaticoduodenectomy remained normogastrinemic after a median follow‐up of 136 months.^26^ Norton et al. demonstrated that surgery may cure ZES in a substantial proportion of patients, with long‐term disease‐free survival achievable in selected cases.^7^, ^31^ The shift toward more aggressive resection with nodal involvement suggests that lymph node metastases are perceived as a critical threshold indicating more aggressive tumor biology.^3^ The reduction in variability between centers in the locally metastatic scenario suggests that nodal involvement represents a clinical inflection point at which institutional experience becomes less influential and a more uniform treatment paradigm emerges.

The relative consistency of management in distant metastatic disease may reflect evidence extrapolated from broader NET populations, particularly the CLARINET and PROMID trials, although direct evidence in MEN1‐associated gastrinomas remains limited.^17^, ^19^ The universal avoidance of PRRT and targeted therapy, despite both being offered as response options, likely reflects the clinical scenario presented: small‐volume, slowly progressive liver metastases in young patients. This is consistent with current ENETS and NCCN guidelines, which reserve these modalities for progressive disease after SSA failure.^3^, ^32^, ^33^ The AACE consensus statement similarly emphasizes that ^177^Lu‐DOTATATE should be reserved for appropriate clinical contexts, such as progressive, somatostatin receptor–positive disease following failure of first‐line somatostatin analogue therapy.^24^ Notably, pivotal trials supporting everolimus, sunitinib, and PRRT have predominantly enrolled patients with pancreatic or non‐functioning GEP‐NETs, which may influence clinician familiarity and lower perceived utility of early systemic escalation in functionally active tumor subtypes such as gastrinoma. These findings demonstrate appropriate clinical judgment across centers and suggest that treatment algorithms for advanced disease are well internalized. The ongoing consideration of curative‐intent surgery in a combined 35.1% of centers reflects the role of surgical approaches in selected patients with limited metastatic burden.^23^

Overall, the observed differences between low‐ and high‐volume centers may have important clinical implications. In the absence of high‐quality evidence supporting a single optimal strategy for non‐metastatic MEN1‐related ZES, management appears to be influenced by institutional experience. High‐volume centers were significantly less likely to recommend immediate surgery and more likely to favor individualized surveillance‐based strategies. Although this study was not designed to assess the superiority of specific approaches, the findings suggest that a conservative strategy may be appropriate in carefully selected patients. Management decisions should therefore be undertaken within experienced multidisciplinary teams, and referral to specialized centers should be considered when local expertise is limited.

The near‐equal division regarding liver biopsy reflects an ongoing clinical debate regarding the necessity of histological confirmation in the context of concordant functional imaging findings. Centers favoring biopsy likely value histological confirmation, Ki‐67 grading, and immunohistochemical characterization to determine the likely primary tumor, particularly given the presence of multiple synchronous lesions of potentially different origin and confirm the biological behavior of the lesions. Centers deferring biopsy may consider the concordant ^68^Ga‐DOTA‐PET/CT findings sufficient for diagnosis and treatment planning while avoiding procedural risk. This variability represents an area where consensus guidance is still needed.

The attribution of metastases predominantly to a duodenal gastrinoma is consistent with the known metastatic potential of duodenal gastrinomas in MEN1.^1^, ^8^ However, the quarter of centers considering a mixed‐origin scenario has important implications for surgical planning, as the perceived primary tumor influences the choice between duodenotomy‐based and pancreas‐focused resection strategies. The integration of transcription factor analysis (ARX, PDX1, CDX2) into pathological evaluation may help resolve this uncertainty.^34^

The preference for MRI as the follow‐up modality is consistent with the 2025 expert guidelines, which recommend MRI as the basis for surveillance of dpNETs.^1^ The selective use of ^68^Ga‐DOTA‐PET/CT reflects its established role in staging and receptor characterization.^11^, ^12^ The relatively short surveillance intervals, with most centers recommending follow‐up every 6 months, reflect the need for close monitoring in metastatic disease.^33^

The universal assessment of standard neuroendocrine markers and Ki‐67 reflects established pathological standards. The variable adoption of advanced molecular markers such as DAXX/ATRX (61.4%), ALT FISH (26.3%), and ARX/PDX1 (35.1%) suggests that integration of these markers into routine practice remains incomplete, despite emerging evidence that they stratify outcomes in pancreatic NETs.^34^ The preferential application of DAXX/ATRX to pancreatic NETs is consistent with the known association of DAXX/ATRX loss with pancreatic rather than duodenal NETs.

This study has several limitations. The use of clinical vignettes may not fully capture the complexity of real‐world decision‐making, and responses reflect intended rather than actual practice. The forced‐choice format may have constrained responses in ways that do not reflect the nuanced deliberations of MDT discussions. Additionally, the absence of outcome data limits the ability to evaluate the effectiveness of different management strategies. The exploratory nature of the statistical analyses, without correction for multiple comparisons, requires cautious interpretation of between‐group differences. Although five participating centers were not ENETS‐accredited Centers of Excellence, their inclusion did not materially influence the principal findings, as demonstrated by a sensitivity analysis restricted to the 52 ENETS Centers of Excellence. The predominant management recommendations across the three clinical scenarios remained unchanged, and the association between lower center volume and a greater preference for surgery in non‐metastatic disease persisted. These findings support the robustness of the observed practice patterns within the participating expert‐center cohort. However, the inclusion of 76.5% of all ENETS CoEs, the participation of 5 additional non‐ENETS expert centers, MDT‐based responses, and a 100% completion rate collectively strengthen the validity and representativeness of the findings.

Future research should focus on prospective studies to define optimal management strategies in non‐metastatic and locally metastatic MEN1‐related ZES. In particular, there is a need to establish evidence‐based surgical thresholds, to define the role of liver biopsy in the metastatic setting, and to optimize integration of molecular markers such as ARX/PDX1 and DAXX/ATRX into clinical decision‐making. The ENETS MEN1 Task Force is well positioned to coordinate such efforts, leveraging the collaborative network established through this study. Development of consensus guidelines informed by both expert opinion and prospective outcome data would help reduce the variability observed in early‐stage disease.^33^

## 5 CONCLUSIONS

Management of MEN1‐related ZES demonstrates clear stage‐dependent variation. While early disease is characterized by substantial heterogeneity and experience‐driven decision‐making, increasing tumor burden leads to convergence toward standardized management strategies. The strong influence of center volume on surgical decision‐making in non‐metastatic disease underscores the importance of multidisciplinary expertise in managing this complex condition. These findings highlight the need for prospective studies and consensus‐driven guidelines to reduce variability and optimize patient outcomes in MEN1‐related ZES.

## AUTHOR CONTRIBUTIONS

**Kosmas Daskalakis:** Conceptualization; methodology; software; data curation; investigation; validation; formal analysis; supervision; visualization; writing – review and editing; writing – original draft; project administration. **Carolina R. C. Pieterman:** Conceptualization; methodology; visualization; writing – review and editing. **Detlef K. Bartsch:** Conceptualization; methodology; investigation; supervision; visualization; writing – review and editing. **Jerena Manoharan:** Writing – review and editing; methodology; data curation. **Simona Grozinsky‐Glasberg:** Methodology; data curation; writing – review and editing; investigation. **James R. Howe:** Methodology; data curation; writing – review and editing; investigation. **Mikkel Andreassen:** Methodology; data curation; investigation; writing – review and editing. **Nehara Begum:** Data curation; investigation; methodology; writing – review and editing. **Dermot O'Toole:** Methodology; data curation; investigation; writing – review and editing. **Timm Denecke:** Data curation; investigation; writing – review and editing. **Antongiulio Faggiano:** Writing – review and editing; methodology; data curation. **Massimo Falconi:** Methodology; data curation; investigation; writing – review and editing. **Stefano Partelli:** Methodology; investigation; data curation; writing – review and editing. **Andreas Pascher:** Methodology; investigation; data curation; writing – review and editing. **Staffan Welin:** Methodology; investigation; writing – review and editing; data curation. **Els Nieveen van Dijkum:** Investigation; methodology; data curation; writing – review and editing. **Alia Munir:** Methodology; investigation; writing – review and editing; data curation. **Aurel Perren:** Methodology; investigation; data curation; writing – review and editing. **Sara Massironi:** Methodology; writing – review and editing; investigation; data curation. **Ana P. Santos:** Methodology; investigation; writing – review and editing; data curation. **Amit Tirosh:** Data curation; investigation; writing – review and editing. **Anna Caterina Milanetto:** Data curation; investigation; writing – review and editing. **Roberta Modica:** Methodology; data curation; investigation; writing – review and editing. **Agnieszka Stefańska:** Investigation; data curation; writing – review and editing. **Guenter Kloeppel:** Methodology; investigation; writing – review and editing. **Piero Ferolla:** Methodology; investigation; writing – review and editing. **Gerlof D. Valk:** Methodology; investigation; writing – review and editing; supervision; visualization; conceptualization; data curation. **Gregory Kaltsas:** Conceptualization; methodology; investigation; data curation; supervision; writing – review and editing.

## CONFLICT OF INTEREST STATEMENT

The authors declare no conflicts of interest.

## Supporting information


**Figure S1.** Distribution of the number of patients with multiple endocrine neoplasia type 1 (MEN1) in active care across the 57 participating expert centers. The x‐axis indicates the number of MEN1 patients in active care. Each blue dot represents an individual center, while the red dot indicates the median number of patients across all participating centers (30 patients).
**Figure S2.** Multidisciplinary team composition across participating centers. Bar chart showing the percentage of centers with each specialty represented in the MDT discussion. Surgeons (94.7%), endocrinologists (89.5%), pathologists (86%), radiologists (86%), nuclear medicine physicians (84.2%), oncologists (84.2%), and gastroenterologists (70.2%).
**Figure S3.** Management of locally metastatic disease stratified by center volume. Grouped bar chart comparing treatment recommendations between low‐volume and high‐volume centers. Surgical management was recommended by 84.6% of low‐volume centers versus 74.2% of high‐volume centers.
**Figure S4.** Management of distant metastatic disease stratified by center volume. Grouped bar chart comparing treatment recommendations between low‐volume and high‐volume centers. Somatostatin analogue therapy was recommended by 50% of low‐volume centers versus 51.6% of high‐volume centers (*p* = .489). Curative‐intent surgery with pancreaticoduodenectomy and liver resection was selected by 23.1% of low‐volume and 29% of high‐volume centers. Curative‐intent surgery with duodenotomy‐based approaches was selected by 15.4% of low‐volume and 3.2%.
**Figure S5.** Imaging modality selection for surveillance in metastatic disease. Bar chart showing the preferred imaging modality among centers (*n* = 50: MRI abdomen (66%), ^68^Ga‐DOTA‐PET/CT (20%), CT abdomen (6%), and other (8%)).

**Data S1.** Complete REDCap questionnaire used in this study with Figure S1. Representative imaging from Vignette 1. MRI abdomen, endoscopic ultrasonography, and ^68^Ga‐DOTA‐PET/CT images demonstrating pancreatic neuroendocrine tumors and duodenal lesions in a patient with MEN1‐related ZES without metastatic disease. Figure S2 shows representative imaging from Vignette 2. MRI abdomen and ^68^Ga‐DOTA‐PET/CT images demonstrating liver metastases and known duodenopancreatic lesions in a patient with MEN1‐related ZES with distant metastatic disease.

## Data Availability

The data that support the findings of this study are available on request from the corresponding author. The data are not publicly available due to privacy or ethical restrictions.
