# Supplementary material for: Stage‐dependent management paradigms for MEN1‐related Zollinger–Ellison syndrome: A vignette‐based study by the ENETS MEN1 Task Force across 57 expert centers
Source: J Neuroendocrinol. 2026 Nov 9;38(11):e70304. doi: 10.1111/jne.70304 (PMC13747676; doi:10.1111/jne.70304)

Vignette February 2025

Resize font:

Please complete the survey below.

Thank you!

1) Please select your institute from the dropdown list

- ◯ Peter MacCALLUM CANCER CENTER
- ◯ Sydney Theranostics (North)
- ◯ Medical University Vienna
- ◯ University Hospital Gasthuisberg-KU Leuven
- ◯ ULB NET Center, Brussels
- ◯ NETwerk
- ◯ Neuroendocrine Tumor Center of Excellence, Rigshospitalet, University of Copenhagen, Depart. PE
- ◯ Aarhus University Hospital ENETS Center of Excellence
- ◯ Hôpital Edouard Herriot
- ◯ Dept of Gastroenterology-Pancreatology, Beaujon Hospital and Paris Diderot University, Clichy
- ◯ IPC Neuroendocrine Tumor Center
- ◯ Universitary Center of Excellence for Neuroendocrine Tumors Toulouse
- ◯ ENETS CoE - Charité, Universitätsmedizin, Berlin, Campus Virchow-Klinikum
- ◯ GEPNET-KUM - Interdisziplinäres Zentrum für Neuroendokrine Tumoren des GastroEnteroPankreatischen Systems des Klinikums der Universität München
- ◯ NET Zentrum Marburg
- ◯ Zentralklinik Bad Berka GmbH
- ◯ Zentrum für Neuroendokrine Tumore, Westdeutsches Tumorzentrum Essen, Klinik für Endokrinologie, Diabetologie und Stoffwechsel, Universitätsklinikum Essen
- ◯ IENET am UCT Universitätsmedizin der Johannes Gutenberg Universität Mainz
- ◯ Zentrum für Neuroendokrine Tumore, Klinikum rechts der Isar
- ◯ Interdisziplinäres Zentrum für Neuroendokrine Tumoren Würzburg
- ◯ NET Centrum Hamburg
- ◯ University hospital of Cologne
- ◯ NET-Zentrum UKSH
- ◯ NET Center Tübingen
- ◯ EKPA-LAIKO CENTER
- ◯ Department of Internal Medicine and Oncology Semmelweis University
- ◯ Neuroendocrine Tumour Centre - St Vincent's Universtiiy Hospital
- ◯ Hadassah-Hebrew University Medical Center
- ◯ National Cancer Institute of Milan
- ◯ Humanitas Research Hospital
- ◯ Multidisciplinary Group for Neuroendocrine Tumors of Naples
- ◯ NET Center Roma Policlinico Agostino Gemelli
- ◯ Sant'Andrea Hospital Sapienza University of Rome
- ◯ Multidisciplinary Group of Verona for Neuroendocrine Tumors
- ◯ European Institute of Oncology

- NET BOLOGNA Multidisciplinary Team
- San Raffaele Hospital Neuroendocrine, Tumor Group
- ENETS centre of excellence Erasmus MC, Rotterdam
- Antoni van Leeuwenhoek/UMC Utrecht, centrum voor neuro-endocriene tumoren
- Amsterdam University Medical Centers
- University Medical Center Groningen
- Center for neuroendocrine tumors, Oslo
- ENETS Center of Excellence, Department of Endocrinology and Neuroendocrine Tumor, Medical University of Silesia, Katowice
- Maria Sklodowska-Curie Memorial Cancer, Centre and Institute of Oncology
- Vall Hebron Institute of Hospital Universitari, Oncology
- Department of Endocrine Oncology Uppsala University Hospital
- University Hospital of Zurich
- Centre des tumeurs neuroendocrines, Lausanne
- Inselspital, Bern
- Universitätsspital Basel - TZ NET
- The Christie Neuroendocrine Centre of Excellence, Manchester
- Liverpool Regional NET Service
- Royal Free Hospital, London
- Imperial College Healthcare NHS Trust, London
- Oxford University Hospitals NHS Trust
- King's Health Partners (Kings College Hospital (lead site), Guys, St Thomas, Kent, North Hampshire Hospital)
- Birmingham Neuroendocrine Tumour Centre
- Sheffield Teaching Hospitals ENETS Centre of Excellence
- Wessex Neuroendocrine Tumour Group
- The ARDEN NET Centre, University, Hospitals Coventry & Warwickshire NHS Trust, WISDEM centre
- West of Scotland NET Centre
- Newcastle Freeman Hospital NET Centre of Excellence
- Belfast NET Centre
- University of Iowa Neuroendocrine Tumor, Center of Excellence
- South Wales Neuroendocrine Cancer Service
- Universitätsklinikum Erlangen; Medizinische Klinik 1, M1 NET-Ambulanz
- Hospital General Universitario Gregorio, Marañón / Hospital Universitario Infanta Sofia, Madrid
- Johannes-Wesling-Klinikum Minden, Minden
- University Hospital of Muenster UKM, Münster
- University Hospital Krakow, Krakow

○ Hospital Universitario 12 de Octubre, Madrid
○ Other

reset

2) If other, please specify

ENETs MEN1 Task Force - Management Questionnaire - Dilemmas in MEN1-related NETs

3) Date vignette discussed in MDT

* must provide value

Today D-M-Y

4) Number of adults MEN1 patients in active care

* must provide value

5) Checklist of MDT-specialties present when case was discussed.

* must provide value

☐ Endocrinologist
☐ Surgeon
☐ Pathologist
☐ Radiologist
☐ Nuclear medicine physician
☐ Oncologist
☐ Gastroenterologist

Case vignette 1:MEN1 and ZES
AIMS of this case-vignette: Gain insight into the management of CoEs for non-metastatic and locally metastatic MEN1-related gastrinoma in ENETS CoEs.

You see a 35 -year-old healthy male in whom MEN1 has been genetically confirmed. He has new biochemically proven symptomatic ZES (reflux, diarrhea) and is normocalcemic. Work-up with gastroduodenoscopy, EUS, 68Ga-DOTA-PET-CT and MRI of the abdomen reveals 3 PanNENs in the pancreatic body and tail 5-15 mm in size, and two small submucosal duodenal lesions < 1 cm, biopsy of one of which reveals a grade I NET with positive staining for gastrin. There is no evidence of lymph node or distant metastases.

Attachment: images case 1.pptx (1.21 MB)

6) How would you treat the patient?

* must provide value

○ Surgically
○ Medically: proton pump inhibitor (PPI) and start somatostatin analogue (SSA)
○ Medically: proton pump inhibitor (PPI) and active surveillance
○ Other

reset

7) If you selected other, please specify

8) If opting for a surgical approach which procedure would be advised?
* must provide value

- ◯ Pancreatico-duodenectomy (Whipple procedure) / pylorus-preserving pancreatico-duodenectomy (Traverso-Longmire procedure)
- ◯ Duodenotomy with excision of duodenal NETs/gastrinomas and regional lymphadenectomy
- ◯ Thompson procedure (left pancreatectomy, duodenal exploration with excision of tumors and node dissection, enucleation of pancreatic head lesions)
- ◯ Pancreas-preserving duodenectomy
- ◯ Total duodenopancreatectomy
- ◯ Other

reset

9) If you selected other, please specify

10) If imaging studies revealed two 68Ga-DOTA-Pet-CT positive lymph nodes near the pancreatic head, how would you then treat the patient?
* must provide value

- ◯ Surgically
- ◯ Medically: proton pump inhibitor (PPI) and start somatostatin analogue (SSA)
- ◯ Medically: proton pump inhibitor (PPI) and active surveillance only
- ◯ Other

reset

11) If you selected other, please specify

12) If opting for a surgical approach which procedure would be advised?
* must provide value

- ◯ Pancreatico-duodenectomy (Whipple procedure) / pylorus-preserving pancreatico-duodenectomy (Traverso-Longmire procedure)
- ◯ Duodenotomy with excision of duodenal NETs/gastrinomas and regional lymphadenectomy
- ◯ Thompson procedure (left pancreatectomy, duodenal exploration with excision of tumors and node dissection, enucleation of pancreatic head lesions)
- ◯ Pancreas-preserving duodenectomy
- ◯ Total duodenopancreatectomy
- ◯ Other

reset

13) If you selected other, please specify

14) Case vignette 1: Open comment box by the MDT (please enter comments) :

Expand

Case vignette 2: Duodenopancreatic (dp) NET with distant metastases
AIMS of this case-vignette:
-Gain insight into the management metastatic dpNETs in MEN1 in the ENETS CoE
-Gain insight into the management of combined ZES and NFpNET in the metastatic setting
-Gain insight into the use of histopathology markers in MEN1-related dpNETs.

You see a 39-year-old patient with genetically proven MEN1 for regular surveillance. This patient is known to have multiple pNETs (the largest 17 mm deep in the pancreatic head and two 5 mm lesions in the body/tail) which have been stable, as well as medically controlled ZES (PPI) with a small duodenal submucosal lesion.
On current surveillance imaging, two liver lesions close to each other of 7 mm and 12 mm (segment 7) are detected (amenable to biopsy), which retrospectively might have been visible previously and have only grown minimally. A 68Ga-DOTA-PET/CT shows uptake confluent to the liver lesions, the known duodenopancreatic lesions as well as two PET-positive peripancreatic lymph nodes (not shown in the attached PET scan).

Attachment: images case 2.pptx (0.19 MB)

15) Would you perform a liver biopsy?
* must provide value

- ◯ Yes
- ◯ No

reset

16) How would you treat the patient?
* must provide value

- ◯ Active surveillance
- ◯ Start somatostatin analogue
- ◯ Curative intent-surgery with pancreatico-duodenectomy (Whipple) and resection of liver lesions
- ◯ Curative intent surgery with duodenotomy, gastrinoma excision, lymphadenectomy and resection of liver lesions
- ◯ Peptide Receptor Radionuclide Therapy (PRRT)
- ◯ Targeted therapy (everolimus, sunitinib)
- ◯ Other

reset

17) If you selected other, please specify

18) If you choose active surveillance, SSA or targeted therapy, when will the next monitoring be done?

- ◯ 3 months
- ◯ 6 months
- ◯ 12months
- ◯ other

reset

19) If you selected other, please specify

20) If you choose active surveillance, SSA or targeted therapy, how would the next monitoring be done (which modality)?

- ◯ Computed Tomography of the abdomen
- ◯ Magnetic Resonance Imaging of the abdomen
- ◯ Endoscopic Ultrasonography of the pancreas
- ◯ 68-Gallium PET CT
- ◯ FDG-PET CT
- ◯ Other

reset

21) If you selected other, please specify

22) **What do you consider to be the most likely primary for the lymph node and liver metastases?**
* must provide value

- ◯ Non-functioning pancreatic NET as primary for both the lymph node and liver metastases
- ◯ Duodenal gastrinoma as most likely primary for both the lymph node and liver metastases
- ◯ Duodenal gastrinoma as most likely primary for the lymph node metastases and NF-PanNET as most likely primary for the liver metastases
- ◯ NF-PanNET as most likely primary for the lymph node metastases and duodenal gastrinoma as most likely primary for the liver metastases
- ◯ Other

reset

23) **If you selected other, please specify** ______

Histopathology
If a liver biopsy was performed, what would you ask for/perform besides Ki-67 and confirmation of metastatic NET. Choose all that apply.

		yes	no
24)	**1. IHC for transcription factors to correlate to primary (TTF-1, Islet-1, PDX1, ARX, CDX2)** * must provide value	☐	☐
25)	**2. IHC for gastrin** * must provide value	☐	☐
26)	**3. SSTR2 immunohistochemistry** * must provide value	☐	☐
27)	**4. other marker**	☐	☐

28) **If you selected other marker, please specify** ______

If an extensive surgical resection has taken place, what would you ask for from the pathologist/would the pathologist determine (choose all that apply)?

		Pancreatic NET	Duodenal NET	Lymph node	Liver metastases
29)	**Synaptophysin/CgA**	☐	☐	☐	☐
30)	**Ki-67**	☐	☐	☐	☐
31)	**DAXX/ATRX**	☐	☐	☐	☐
32)	**ALT Fish**	☐	☐	☐	☐
33)	**ARX/PDX1**	☐	☐	☐	☐
34)	**IHC gastrin**	☐	☐	☐	☐
35)	**IHC insulin**	☐	☐	☐	☐
36)	**IHC glucagon**	☐	☐	☐	☐
37)	**Other**	☐	☐	☐	☐

38) **If you selected other, please specify** ______

39) **Case vignette 2: Open comment box by the MDT (please enter comments) :** ______

Expand

Submit

Powered by REDCap

Supplementary Figures S1 (1st case) and S2 (2nd case)

Figure S1a

35 year-old male, pH in stomach = 1, basal gastrin 458pg/ml (normal<125pg/ml)

Duodenoscopy : 2 submucosal lesions <10mm in diameter (arrows)

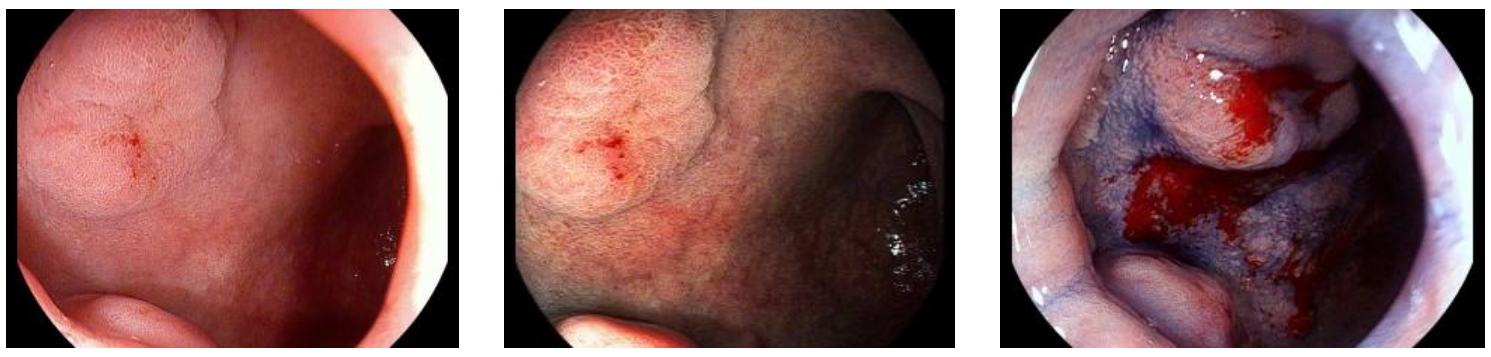

Figure S1b

MRI abdomen - 3 pNET 5-15mm body/tail (arrows)

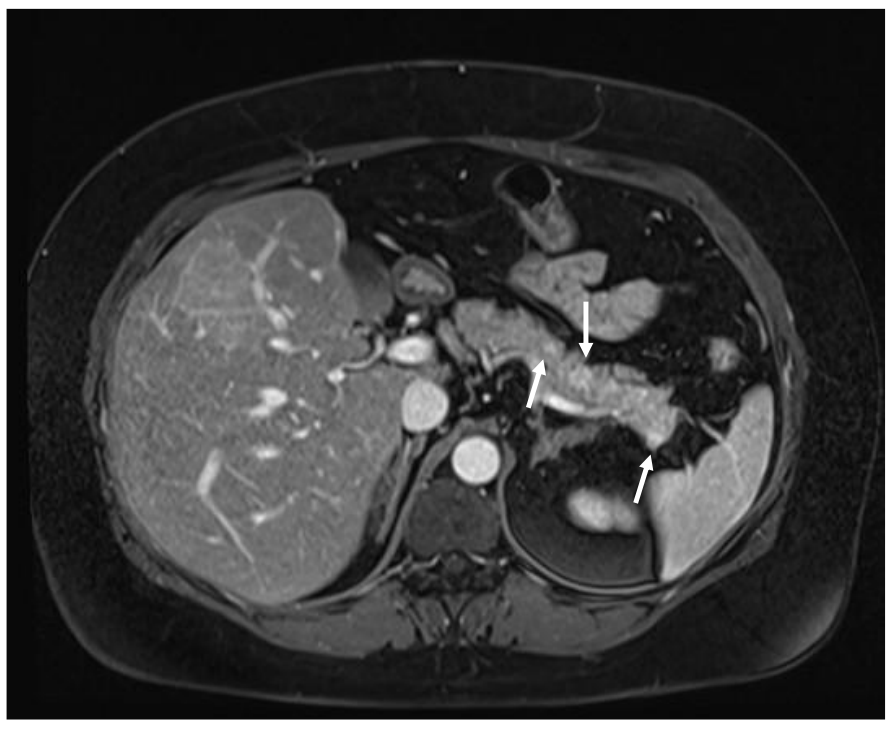

Figure S1c

Ga68 PET/CT – 2 positive lesions in body/tail (arrows)

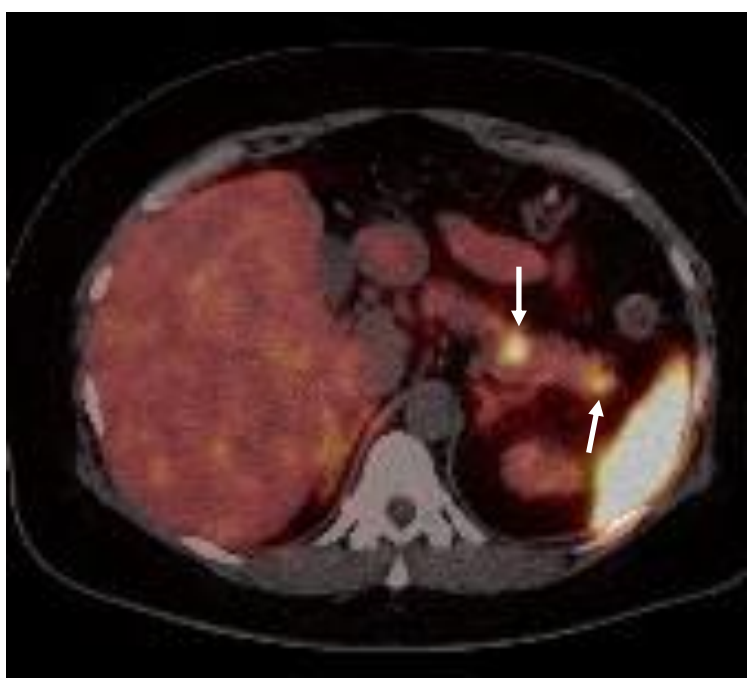

Figure S2a

39 year-old male with multiple pNETs and ZES with a small submucosal lesion in the duodenum.

MRI abdomen- 2 NELMs 7 and 12mmin size in liver segment 7

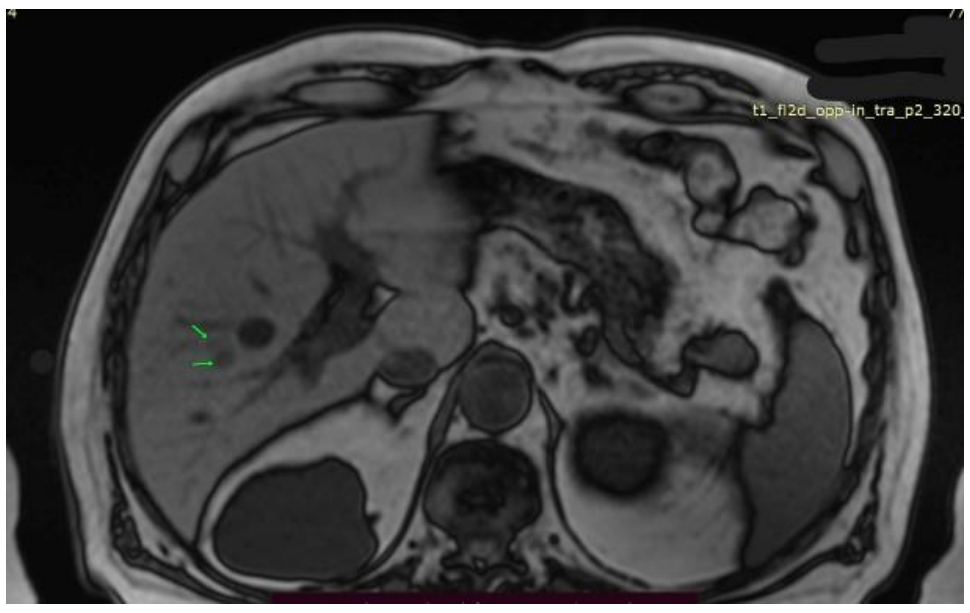

Figure S2b

Ga68 PET/CT – uptake in 2 positive lesions close to each other in liver segment 7(arrow)

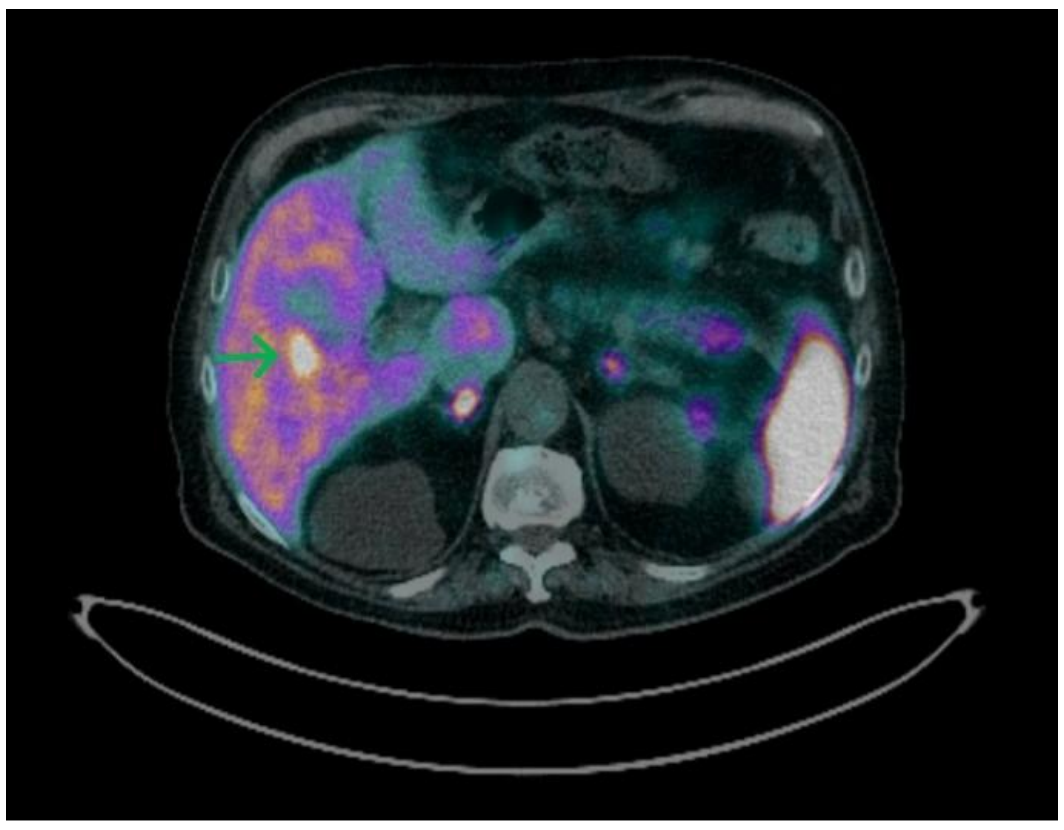

Supplement: Supplementary file 1 [file JNE-38-e70304-s001.pdf]
