# Supplementary material for: Stage‐dependent management paradigms for MEN1‐related Zollinger–Ellison syndrome: A vignette‐based study by the ENETS MEN1 Task Force across 57 expert centers
Source: J Neuroendocrinol. 2026 Nov 9;38(11):e70304. doi: 10.1111/jne.70304 (PMC13747676; doi:10.1111/jne.70304)
Supplement: Supplementary file 2 [file JNE-38-e70304-s002.docx]

**Supplementary Apendix 2**

**Figure S1** Distribution of the number of patients with multiple endocrine neoplasia type 1 (MEN1) in active care across the 57 participating expert centres. The x-axis indicates the number of MEN1 patients in active care. Each blue dot represents an individual centre, while the red dot indicates the median number of patients across all participating centres (30 patients).

**Figure S2.** Multidisciplinary team composition across participating centres. Bar chart showing the percentage of centres with each specialty represented in the MDT discussion. Surgeons (94.7%), endocrinologists (89.5%), pathologists (86%), radiologists (86%), nuclear medicine physicians (84.2%), oncologists (84.2%), and gastroenterologists (70.2%).

**Figure S3.** Management of locally metastatic disease stratified by centre volume. Grouped bar chart comparing treatment recommendations between low-volume and high-volume centres. Surgical management was recommended by 84.6% of low-volume centres versus 74.2% of high-volume centres (χ^2^, p=0.336).

**Figure S4.** Management of distant metastatic disease stratified by centre volume. Grouped bar chart comparing treatment recommendations between low-volume and high-volume centres. Somatostatin analogue therapy was recommended by 50% of low-volume centres versus 51.6% of high-volume centres (p=0.489). Curative-intent surgery with pancreatico-duodenectomy and liver resection was selected by 23.1% of low-volume and 29% of high-volume centres. Curative-intent surgery with duodenotomy-based approaches was selected by 15.4% of low-volume and 3.2%

**Figure S5.** Imaging modality selection for surveillance in metastatic disease. Bar chart showing the preferred imaging modality among centres (n = 50: MRI abdomen (66%), ^68^Ga-DOTA-PET/CT (20%), CT abdomen (6%), and other (8%).
